# A novel approach to reverse Warburg metabolism in patients with recurrent glioblastoma: A phase II pharmacodynamic study of dichloroacetate

**DOI:** 10.1093/noajnl/vdag223

**Published:** 2026-08-25

**Authors:** Carolyn O Dirain, Stuart A Grossman, Xiaobu Ye, Priya Sambasivan, Puranjay Shori, Himashi Liyanarachchi, Thomas D Franklin, Joy Fisher, Trisha Surakus, Michaella Iacoboni, Matthias Holdhoff, Karisa C Schreck, Stephen B Tatter, Richard E Wagner, Roy Strowd, Chetan Bettegowda, Peter W Stacpoole

**Affiliations:** Department of Medicine, University of Florida College of Medicine, Gainesville; Department of Oncology, Sidney Kimmel Cancer Center, Johns Hopkins University School of Medicine, Baltimore; Department of Oncology, Sidney Kimmel Cancer Center, Johns Hopkins University School of Medicine, Baltimore; Department of Medicine, University of Florida College of Medicine, Gainesville; Department of Medicine, University of Florida College of Medicine, Gainesville; Department of Medicine, University of Florida College of Medicine, Gainesville; Department of Medicine, University of Florida College of Medicine, Gainesville; Department of Oncology, Sidney Kimmel Cancer Center, Johns Hopkins University School of Medicine, Baltimore; Department of Oncology, Sidney Kimmel Cancer Center, Johns Hopkins University School of Medicine, Baltimore; Department of Oncology, Sidney Kimmel Cancer Center, Johns Hopkins University School of Medicine, Baltimore; Department of Oncology, Sidney Kimmel Cancer Center, Johns Hopkins University School of Medicine, Baltimore; Departments of Neurology and Oncology, Johns Hopkins University School of Medicine, Baltimore; Department of Neurosurgery, Comprehensive Cancer Center, Wake Forest School of Medicine, Winston-Salem; Medosome Biotec LLC, Alachua; Departments of Neurology, Internal Medicine, and Translational Sciences Institute, Wake Forest School of Medicine, Winston-Salem; Department of Neurosurgery, Johns Hopkins University School of Medicine, Baltimore; Department of Medicine, University of Florida College of Medicine, Gainesville; Department of Biochemistry and Molecular Biology, University of Florida College of Medicine, Gainesville

**Keywords:** dichloroacetate (DCA), glioblastoma (GBM), lactate, pyruvate dehydrogenase complex (PDC), pyruvate dehydrogenase kinase (PDK), Warburg metabolism

## Abstract

**Background:**

Glioblastomas are characterized by the Warburg effect, driven by upregulation of pyruvate dehydrogenase kinase (PDK), which inhibits pyruvate dehydrogenase complex (PDC), leading to lactate accumulation. Dichloroacetate (DCA) is a potent and safe PDK inhibitor that crosses the blood-brain barrier, reverses Warburg metabolism, and reduces lactate levels.

**Methods:**

This trial (RO1FD007271) evaluated the pharmacodynamics and pharmacokinetics of oral DCA in recurrent glioblastoma patients requiring surgical debulking. The primary endpoint was decreased PDC phosphorylation (p-PDHA1) in resected tumors. Patients received either 1 week of DCA or no DCA prior to surgery. All patients received DCA postoperatively. Enhancing and non-enhancing tumor tissue, and serial plasma DCA and lactate levels were analyzed.

**Results:**

37 patients were enrolled (median age = 60 years). In DCA-treated patients, the contrast-enhancing tumor had lower p-PDHA1, PDK4, HIF1-α, VEGF-α, and PGK1 expression (all *P* < .05) than non-DCA-treated patients. In non-enhancing tumors, p-PDHA1 and PDK 1-3 expression were not different, but PDK4, PCNA, and PGK1 levels were reduced, and ERK1/2 was increased in DCA-treated patients (all *P* ≤ .01). At surgery, DCA-treated patients had lower plasma lactate (*P* = .004) than untreated patients. Postoperatively when all patients received DCA, plasma lactate fell dramatically (*P* < .001). DCA was well-tolerated but did not delay tumor recurrence.

**Conclusions:**

In recurrent glioblastomas, DCA was safe, well-tolerated, and promoted aerobic respiration. It reduced markers of tumor cell proliferation and lowered plasma lactate. Although no clinical benefit was noted, further studies of combination therapy are indicated, given the known association between poor cancer outcomes and elevated PDK expression and lactate levels.

Key Points

- Chronic oral DCA was well tolerated and safe in adults with recurrent GBM.
- DCA resulted in dramatic reductions in plasma lactate and the reversal of Warburg metabolism in resected tumor by inhibiting PDK-induced PDC phosphorylation and reducing tumor proliferation markers.

Importance of Study

Glioblastoma (GBM) is the most common and aggressive primary brain cancer in adults. Left untreated, mean life expectancy of newly diagnosed patients is 3-4 months. Current standard of care (SOC) treatment with surgery, radiation, and chemotherapy (temozolomide, TMZ) extends life only approximately 16 months and has no curative potential. There are no current options for prevention or early diagnosis of these tumors, and there has been minimal change in overall survival over the past several decades. Dichloroacetate (DCA) is a small molecule that readily crosses the blood-brain barrier. DCA inhibits pyruvate dehydrogenase kinase (PDK) that inhibits the mitochondrial pyruvate dehydrogenase complex (PDC) by reversible phosphorylation. Pharmacological inhibition of PDK in cancer cells by DCA restores PDC activity, reverses Warburg metabolism, and induces a caspase-mediated selective apoptosis of tumors. Extensive preclinical research and early clinical trials indicate that DCA may be a safe and uniquely effective metabolic therapy for GBM.

Glioblastoma (GBM) is the most common and aggressive primary brain cancer in adults.^1^ Left untreated, mean life expectancy of newly diagnosed patients is 3-4 months. The current standard of care (SOC) treatment with surgery, radiation, and chemotherapy (temozolomide, TMZ) extends life only approximately 16 months and has no curative potential.^2^ Less than 5% of patients survive longer than five years. There are no current options for prevention or early diagnosis of these tumors, and there has been minimal change in overall survival (OS) over the past decades.

A cardinal metabolic characteristic of GBM, and cancer in general, is a metabolic rewiring in tumors that favors glycolysis over oxidative phosphorylation (OXPHOS), a phenomenon known as the Warburg effect.^3^ As a consequence, tumor metabolism is often characterized by accelerated glycolysis, even in the presence of adequate tissue oxygenation, leading to markedly increased production of lactate, a molecule that fosters immunosuppression and metastatic spread in cancers.^4^ The glycolytic shift that occurs in GBM is mechanistically associated with post-translational inhibition of the mitochondrial pyruvate dehydrogenase complex (PDC), which normally catalyzes the rate-limiting step in the aerobic oxidation of glucose-derived pyruvate and lactate to acetyl coenzyme A (AcCoA). PDC inhibition is due to transcriptional upregulation of 1 or more of 4 differentially expressed pyruvate dehydrogenase kinase isoforms (PDK 1-4) that inhibit PDC by reversible phosphorylation. Pyruvate is in equilibrium with lactate via lactate dehydrogenase (LDH); therefore, the PDC also regulates cellular oxidative lactate removal.

Elevated blood or tissue lactate is a hallmark of human disease,^5^ and it is impossible to dissociate the impact of lactate metabolism and the development and progression of tumors. Indeed, due to the combined effects of accelerated glycolysis (Warburg metabolism) and PDK-mediated PDC inhibition, intratumoral lactate concentrations may exceed 10-20 mM and have been inversely associated with tumor recurrence, proliferation, and survival in diverse human cancers.^3^^,^^6^ Magnetic resonance imaging studies of GBMs have revealed high lactate concentrations relative to normal brain tissue and an inverse association between tumor lactate and survival.^7^ However, blood or tumor lactate concentrations may be barely elevated above normal (1mmol/l) to portend increased mortality.^6^

Dichloroacetate (DCA) is a small molecule and investigational drug that is readily transported across cell membranes in non-tumor tissue, including the blood-brain barrier (BBB) in animal studies, via the monocarboxylate transport system and enters mitochondria through the pyruvate carrier system.^3^ BBB transport of DCA in humans has not been reported. It is a structural analog of pyruvate that can occupy the pyruvate binding site on PDK, inhibiting its activity and maintaining the PDC in its unphosphorylated, active form.^8^ Chronic exposure to DCA also results in stabilization of the E1a subunit (PDHA1) of the complex,^9^^,^^10^ thereby retarding PDC turnover. This dual mechanism of activation of the PDC by DCA accounts for the drug being the most potent lactate-lowering agent used clinically.^11^ Inhibition of PDKs by DCA restores PDC activity and reverses Warburg metabolism in cancer cells.^3^ In so doing, it reduces tissue lactate concentrations in both the tumor microenvironment (TME) and the blood.

DCA inhibits its own metabolism, and its only clinically limiting toxicity is a reversible peripheral neuropathy.^12^ To mitigate or prevent this adverse effect, we developed and validated a genotyping method for genetics-based dosing of DCA that dichotomizes subjects into fast and slow DCA metabolizers based on haplotype variation in glutathione transferase zeta 1 (GSTZ1), leading to safe, personalized DCA dosing.^13^ Employing this genetics-based dosing, we have shown that chronic oral DCA is safe in a prior study of patients with recurrent brain tumors.^14^ In addition, we recently completed a phase 3 multicenter trial of oral DCA in young children with congenital PDC deficiency (PDCD) and found that DCA is well tolerated and safe, significantly decreases plasma lactate concentrations, and improves survival in children with PDCD.^15^

The primary objectives of this study were: (1) to compare the phosphorylated (inactive) PDC (p-PDHA1) protein level in tumor tissue between patients with GBM who were treated with or without DCA for one week prior to surgical resection of the tumor and (2) to assess toxicity of personalized DCA dosing per individual genotype during the DCA treatment. The secondary objectives were to (1) compare intratumoral biochemical correlates in tumor tissue excised from DCAtreated and DCA-non-treated subjects and (2) to estimate the progression-free survival (PFS) and OS in all post-surgical subjects receiving DCA.

## Methods

### Trial Design and Participants

This multicenter, open-label, Phase IIA trial was funded by the Orphan Products Division of the Food and Drug Administration (RO1FD007271) and Saol Therapeutics to determine if orally administered DCA is well-tolerated, safe, and results in the desired pharmacodynamic effects on surgically resected recurrent glioblastomas. The trial was reviewed and approved by the Institutional Review Boards of the University of Florida (IRB202101509, IRB of record), Johns Hopkins University, and Wake Forest University. The trial is registered with clincialtrials.gov under NCT05120284.

**Figure 1.**
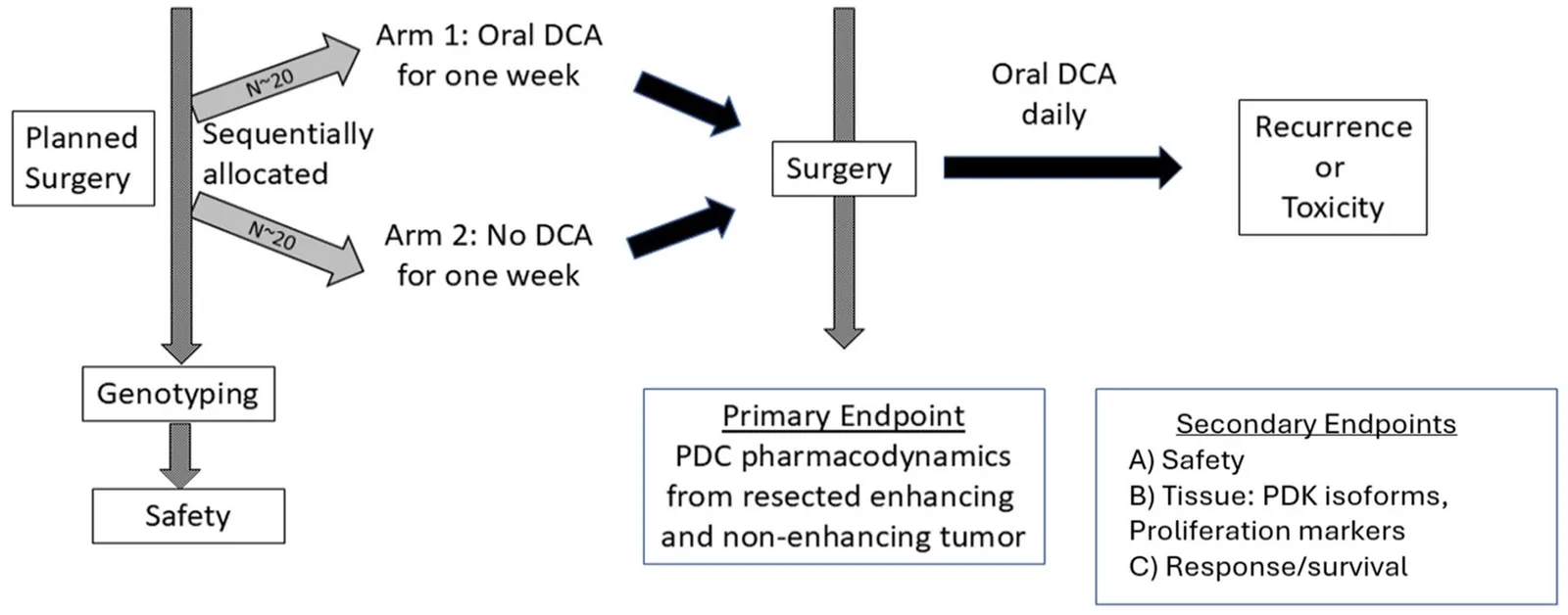
Study design of phase 2A trial.

A total of 40 patients with recurrent GBMs who had clinically indicated debulking surgery planned were to be accrued to this study. Patients in each treatment arm were genotyped and sequentially allocated to receive DCA or no DCA for 1 week prior to surgery (Figure 1). Those allocated to receive DCA received 12.5mg/kg of DCA every 12 hours for the week prior to surgery. At the time of surgery, tissue that was not required for pathologic analysis from both enhancing and non-enhancing tumors was processed as outlined below to meet the primary study endpoints. Approximately 1 month postoperatively, all patients were offered DCA postoperatively at a dose determined by the results of their GSTZ1 haplotype analysis. “Fast” drug metabolizers (EGT carriers) were dosed at 12.5 mg/kg every 12 hours while “slow” metabolizers (EGT noncarriers) were dosed at 6.25 mg/kg DCA every 12 hours. Data on safety, response, survival and plasma DCA and lactate levels were collected serially.

### Patient Population

Patients eligible for this study were required to be between 18 and 80 years old with a histologically confirmed glioblastoma, and have previously received standard radiation and TMZ, have progressed radiographically and clinically, and require clinically indicated debulking surgery. Standard neurosurgical neuronavigational techniques were used to identify, sample, and record enhancing and non-enhancing regions of the tumor. However, specific imaging requirements, clinical evidence of tumor progression, or a required interval from completion of radiation therapy were not required. In addition, they must have recovered from toxicities of prior therapies and have a Karnofsky Performance Status of 60 or higher, with normal hematologic and organ function. Patients who were pregnant, receiving other anticancer therapies for their glioblastoma, or unable to provide informed consent or swallow liquids were excluded from this trial, as were patients with diabetes requiring insulin or sulfonylurea. The full eligibility criteria are available in the supplementary material that accompanies this manuscript.

### Pharmacodynamic Tissue Analysis Endpoints

The primary efficacy endpoint was a statistically significant decrease in the mean p-PDHA1 in surgical tissue from subjects who received DCA for the week before debulking surgery vs. mean p-PDHA1 expression in the tissue of subjects not receiving presurgical DCA, ie DCA would reach tumor tissue in sufficient concentration to achieve an expected pharmacodynamic effect. Based on extensive preclinical studies of the impact of DCA on PDC activity and expression, we considered this to be a realistic and readily achievable objective.

Tumor tissues (T1, contrast-enhanced, and T2, non-contrast-enhanced) obtained at surgery were analyzed for expression of total and phosphorylated PDHA1, PDK isoforms 1-4 and several markers of tumor proliferation. After recovering from debulking surgery, all patients received DCA. Plasma samples were collected before, during, and after surgery to measure DCA and lactate levels by gas chromatography/mass spectrometry.

### Pharmacodynamic Tissue Analyses

#### Protein Expression.—

Protein expression of phosphorylated PDHA1 (p-PDHA1), total PDHA1, PDK 1 to 4, PDH phosphatase-1 (PDP1), and tumor markers Hypoxia Inducible Factor-1 alpha (HIF1-α), Vascular Enhancing Growth Factor-alpha (VEGF-α), Proliferating Cell Nuclear Antigen (PCNA), phosphoglycerate kinase 1 (PGK1), and Extracellular Signal Regulated Kinase 1 and 2 (ERK 1/2), in enhancing and non-enhancing GBM tissue lysates, was evaluated by Western blotting. T1 and T2 samples were analyzed separately.

Briefly, tumor samples were pulverized in liquid nitrogen. Except for HIF1-α, cell proteins were extracted using RIPA lysis buffer with protease and phosphatase inhibitor cocktail (Thermo-Fisher Scientific, Waltham, MA, USA). For HIF1-α, cytoplasmic and nuclear proteins were extracted using Nuclear Extract Kit (ab219177, Abcam Inc., Waltham, MA, USA). Protein concentrations were determined (BCA Protein Assay, Thermo-Fisher Scientific, Waltham, MA, USA), and samples were normalized according to protein concentration.

Tissue lysate was mixed with an equal volume of 2× Laemmli buffer with 5% 2-mercaptoethanol. Twenty microliter samples (15 or 20 μg of protein) were loaded per lane in 4%-20% TGX™ Stain-Free gel, separated by SDS-PAGE and transferred onto a nitrocellulose membrane (BioRad Laboratories, Hercules, CA, USA). The membrane was blocked with 5% BSA/1xTBS with 0.05% Tween-20, or 5% nonfat milk/0.05% Tween-20 for HIF1α for 1 hour and then incubated in primary antibody overnight at 4 °C. The following primary antibodies were used: PDHA1, PDK3, PDK4, and HIF1-α (Invitrogen, Waltham, MA, USA); p-PDHA1, PDK1, VEGF-α, and PDP1 (Cell Signaling Technology, Danvers, MA, USA); PDK2, PCNA, and ERK1/2 (Santa Cruz Biotechnology, Dallas, TX, USA); and PGK1 (Signalway Antibody, College Park, MD, USA). The secondary antibodies were Starbright 700 and Starbright 520 (Bio-Rad Laboratories, Hercules, CA, USA). The membrane was washed with TBS/0.05%Tween-20 and then incubated with the corresponding secondary antibody. The membrane was washed, then protein bands were visualized (ChemiDoc MP Imaging System), and the band intensities were quantified (ImageLab software, BioRad Laboratories, Hercules, CA, USA). The stain-free blot image was used for total protein loading control and normalization, ie lane density of total protein was used to normalize target protein band density.

Since tumor samples were analyzed as they became available and only 10-11 samples can be loaded in one gel, Western blotting was done in batches. To normalize data within each gel and between gels, we employed the relative normalized ratio (RNR) in Western blotting as previously described.^16^

#### *Plasma DCA and Lactate Levels*.—

Levels of DCA and lactate in plasma from the GBM patients were measured by GC-MS.^17^ Calibration standards were also prepared by adding known amounts of DCA (TCI America, Portland, OR, USA) and lactate (Sigma-Aldrich, St. Louis, MO, USA) to human plasma from a blood bank (LifeSouth, Gainesville, FL, USA) and derivatized and analyzed like the GBM plasma samples. Analytical blanks (HPLC water, Thermo Fisher Scientific, Waltham, MA, USA) were also prepared. Blanks, standards, and samples were run in duplicate in accordance with good laboratory practice. The internal standard, 2-oxohexanoic acid (OHA, Sigma-Aldrich, St. Louis, MO, USA), was added to all samples, blanks, and standards before derivatization and extraction. Briefly, a plasma sample (200 μL) and the internal standard (OHA, 500 μg/mL in water, 50 μL) were mixed with 500 μL of 12% BF_3_–MeOH in a sealed glass culture tube by vortexing for 5 min. The mixture was heated at 95 °C for 20 min. After cooling, 1 mL of methylene chloride and 1 mL of HPLC water were added to the reaction solution. The sample was vortexed for 5 min and centrifuged (3000 rpm, 10 °C for 15 min) to separate the layers. The lower methylene chloride layer was transferred to an autosampler vial for GC/MS analysis.

Extracted samples were analyzed using an Agilent 5977 GC-MS (Agilent Technologies, Palo Alto, CA) under analytical conditions previously reported.^17^

### Statistical Analysis

#### *Sample Size Determination*.—

The primary efficacy endpoint in this study was the protein level of phosphorylated PDHA1 (pPDHA1) in surgically resected GBM tissue. The primary study hypothesis states that patients treated with DCA prior to the surgery will have increased tumor PDC activity by decreasing pPDHA1 on an average of 50% or greater in tumors from patients who were treated with DCA than those who were not. Assuming the standard deviation on its measurement scale is no larger than 0.4, 20 patients per arm (treated and non-treated) would yield 90% power to detect an average of 50% difference at a significant level of 0.05 (2-sided). The design required a minimum of 50% of patients to reach a 50% reduction in pPDHA1 after 1 week of DCA treatment compared to the reference untreated group to be considered successful. The sample size was calculated to provide 94% power to detect a 50% success rate, compared to a null of 15% at a 2-sided significant level of 0.05.^18^

Biochemical data are expressed as the mean ± SE. Each protein was treated as an independent, pre-specified outcome rather than being analyzed with a multiple testing framework. Two-sided Student’s *t*-test was used to assess differences between treatment groups for each protein. Statistical tests were performed using JMP Pro 18 software (SAS Institute, Cary, NC, USA). A *P*-value ≤ .05 was considered statistically significant.

### Safety Analysis Endpoints

The second primary objective of the study was to assess the toxicity of DCA in this patient population. The NC/I Common Terminology Criteria for Adverse Events (CTCAE) version 5.0 was used for scoring toxicity and adverse events. The primary safety endpoint was the degree of dose-limiting toxicity, defined as a grade 3 or greater clinically significant AE or abnormal laboratory value assessed as unrelated to disease progression, intercurrent illness, or concomitant medications. The severity and frequency of toxicity were tabulated by the tested dose or doses using descriptive statistics. The proportion of subjects who experienced grade 3 or above toxicities was estimated, along with 95% confidence intervals by each type of toxicity. Dose-limiting toxicity was used in analyzing the safety endpoint. A Data Safety Monitoring Committee at Johns Hopkins comprised of Drs. Mario Eisenberger, John Laterra, and Raj Mukherjee reviewed safety data throughout the course of the study.

### Secondary Endpoints

A secondary objective was to estimate the PFS and OS in all patients receiving post-surgical DCA. Progression was defined by any of the following: (a) ≥ 25% increase in sum of the products of perpendicular diameters of enhancing on stable or increasing doses of corticosteroids; (b) a significant increase in T2/FLAIR non-enhancing lesion on stable or increasing doses of corticosteroids; (c) the appearance of any new lesion; (d) clinical deterioration not attributable to other causes; (e) failure to return for evaluation due to death or deteriorating condition; or (f) clear progression of non-measurable disease. Survival time was defined from the date of study registration to the date of death. The Kaplan-Meier method was used to estimate median OS, together with a 95% confidence interval.

## Results

### Patient Population and Accrual Information

The original plan had been to accrue 40 patients. However, accrual was stopped prematurely at 37 patients due to lack of drug supply. Of the 37 subjects enrolled, 2 had their planned surgery canceled because their medical team felt this was no longer the best therapeutic option for their recurrent glioblastoma. They never received DCA. A third was found to have an ineligible histology after enrollment. As a result, 34 patients (28 from Johns Hopkins University and 6 from Wake Forest University) are included in the analysis. Patient demographics are presented in Table 1. Participants were a median age of 60 years; 76% were white, 71% were male, and 67% had a KPS of >90 (Table 1).

**Table 1.** Demographics and clinical data of GBM study participants

Patient baseline characteristics	All patients (*N* = 34)
Age: Median (Range)	60 (43-74)
Race:	
White: No. (%)	26 (76%)
Gender: Male No. (%)	24 (71%)
KPS:	
90-100: No. (%)	22 (67%)
70-80: No. (%)	11 (33%)
60	1 (3%)
Histology prior study:	
Glioblastoma Multiforme (IV): No. (%)	34 (100%)
IDH Wildtype: No. (%)	34 (100%)
Surgery:	
Biopsy: No. (%)	1 (3%)
Subtotal Resection: No. (%)	17 (50%)
Gross Total Resection: No. (%)	16 (47%)
Number of Prior surgeries: median (range)	1 (1-2)
Prior treatment: No. (%)	
1 prior treatment (TMZ + RT (SOC) +/− another drug)	34 (100%)
2 prior treatments (SOC, relapse 2^nd^ drug)	4 (12%)

### Analysis of Surgically Resected Glioblastoma Tissue

Of the 34 patients who underwent resection surgery, viable tumor tissue suitable for biochemical analysis was obtained from the contrast-enhancing tumor in 26 patients. Thirteen of these patients received preoperative DCA and 13 did not receive preoperative DCA. Viable non-enhancing tumor tissue was also obtained from 24 patients. Twelve of these patients had received DCA preoperatively, and 12 did not receive preoperative DCA. This reduction was due to early termination of the trial related to drug supply constraints and availability of evaluable tissue samples. The reduced sample size may limit precision of effect estimates and the ability to detect more modest differences. But despite this reduced analyzable sample, significant differences were observed between the treatment groups in the pre-specified primary endpoint as well as in several additional protein markers. These findings suggest a treatment effect that was detectable even within the smaller sample size.

**Figure 2.**
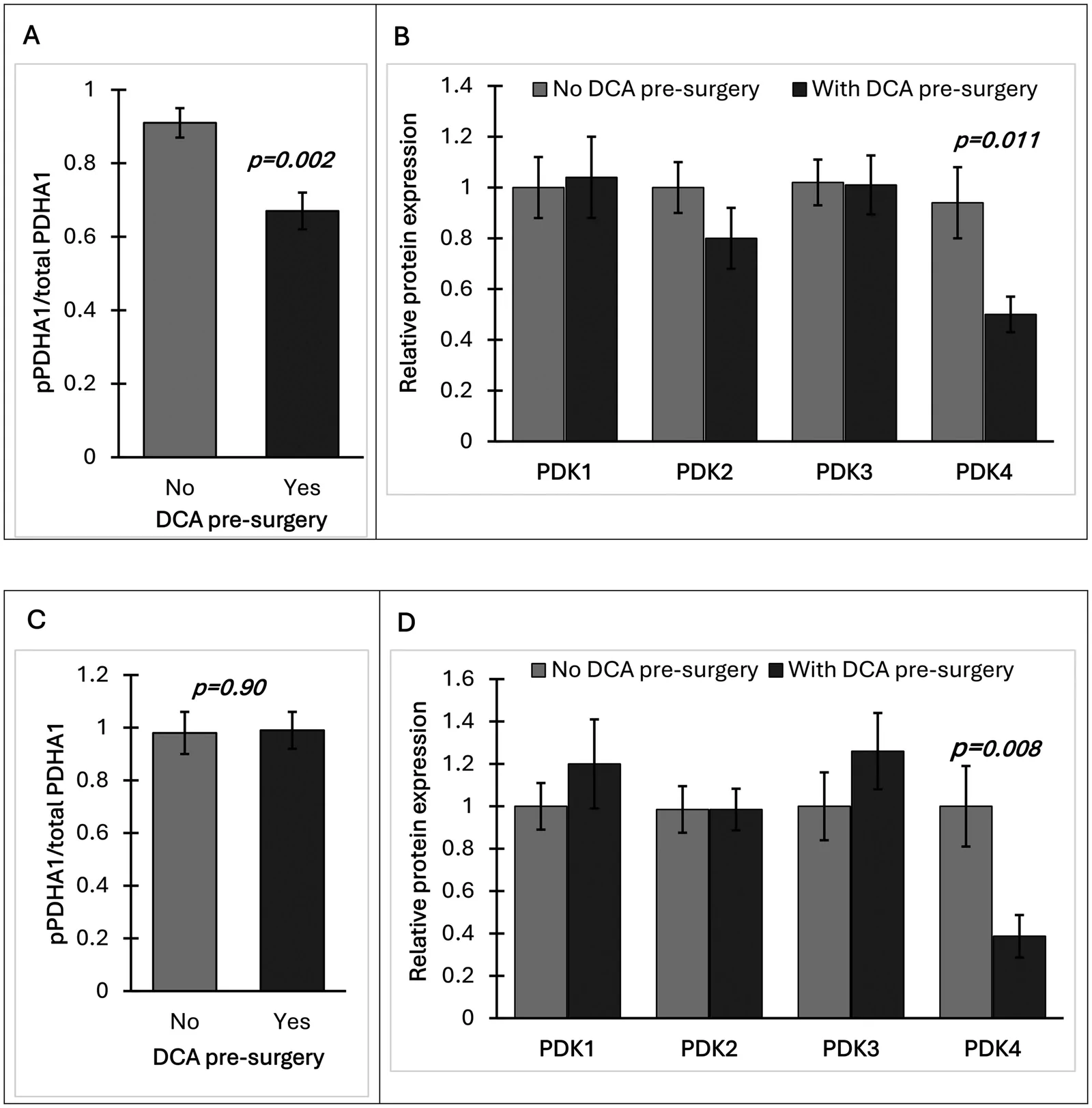
Effect of DCA on PDC phosphorylation (A) and PDK1-4 expression (B) in contrast-enhancing tissues and on PDC phosphorylation (C) and PDK1-4 expression (D) in non-contrast-enhancing tissues.

Biochemical analysis showed lower p-PDHA1 (*P* = .002) and PDK4 (*P* = .011) in the more necrotic (contrast-enhancing) tumors of patients who received DCA pre-surgery (*n* = 13), compared to patients without DCA (*n* = 13) (Figure 2A, B). These findings are consistent with the known action of DCA and suggest a shift toward aerobic respiration. PDK4 was also lower in the DCA pre-surgery group in the non-enhancing tumor tissue (*P* = .008), but there were no differences in p-PDHA1 or PDK 1-3 expression (Figure 2C, D). There were no differences in PDP1 (data not shown) and total PDHA1 expression in either contrast-enhancing or non-contrast-enhancing tissues, again, in keeping with the lack of effect of DCA on total PDC and PDP1 expression in any tissue, normal or malignant.^3^

**Figure 3.**
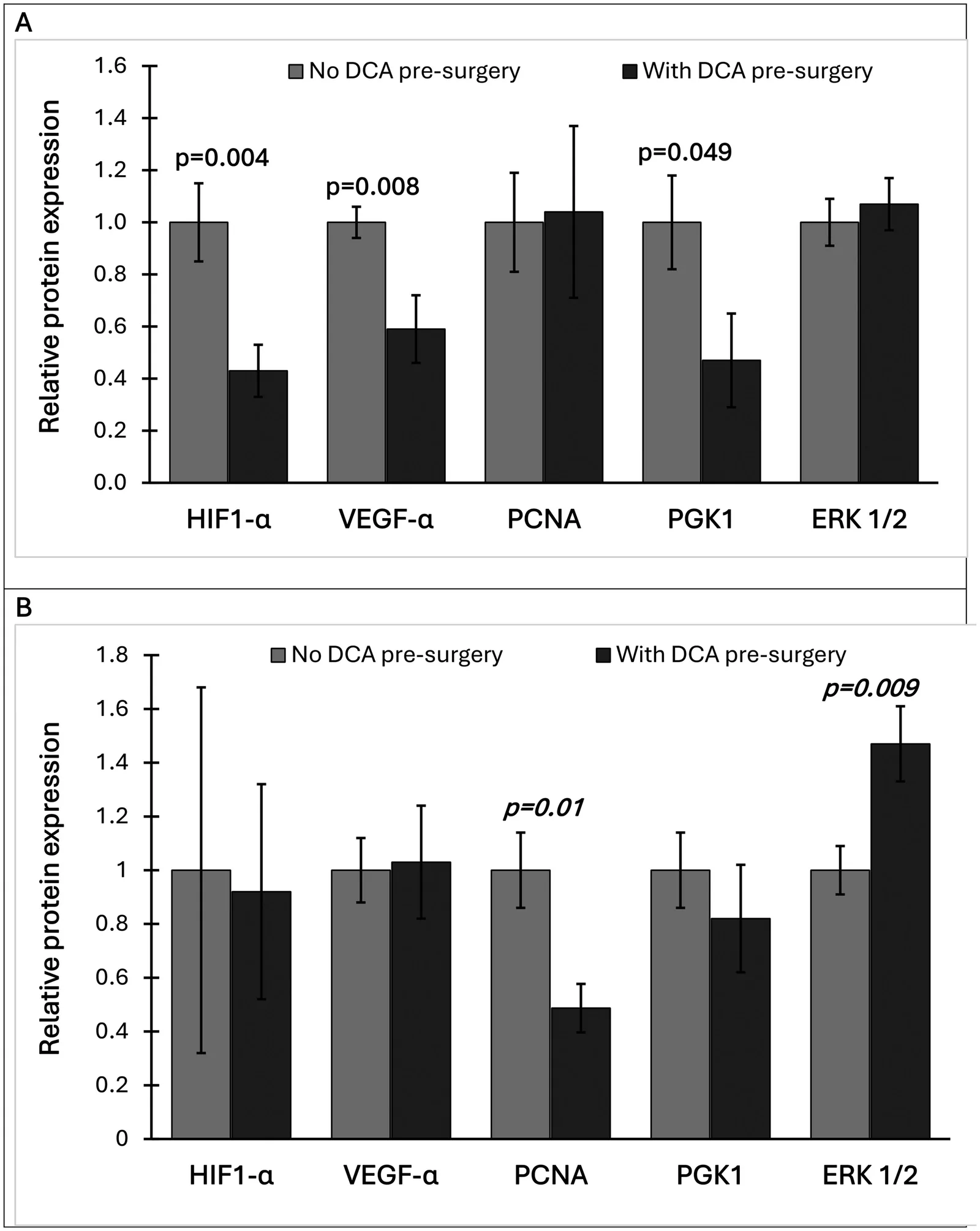
Effect of DCA on tumor markers in contrast-enhancing (A) and non-contrast enhancing (B) tissues.

We next examined expressions of several known tumor markers associated with cellular proliferation. Intriguingly, in DCA-treated patients, the nuclear expression of HIF-1α, a master transcription factor that enhances expression of several glycolytic genes and also mitochondrial PDKs, thus promoting the ability of cancer cells’ adaptation to hypoxic environments and a protein that is stabilized by lactate,^19^ was significantly reduced in enhancing GBM tissue (*P* = .004) (Figure 3A). VEGF-α, a protein that stimulates tumor angiogenesis and vascular proliferation characteristic of high-grade gliomas,^20^ and PGK1, an essential enzyme in aerobic glycolysis, high expression of which promotes tumor cell proliferation and invasion,^19^ were also reduced (*P* = .049, Figure 3A) in contrast-enhancing tissue. In the non-enhancing tumors, PCNA, a marker also used to evaluate cell proliferation and tumor prognosis^21^ was significantly reduced (*P* = .01), and ERK 1/2, reported to play a crucial role in cell proliferation and migration of glioma cells and to induce apoptosis upon sustained activation^22^ was increased in patients who received DCA pre-surgery (*P* = .009, Figure 3B). Together, these changes suggest that DCA may decrease tumor proliferation.

### Analysis of Plasma Lactate Levels

Thirty-three patient plasma samples at baseline (*n* = 16 without DCA pre-surgery and *n* = 17 with DCA pre-surgery) were analyzed for lactate levels. In the beginning of the trial, plasma samples were not collected during surgery in patients who did not receive DCA pre-surgery, with the assumption that their plasma lactate would be the same at baseline and surgery. The protocol was changed, and plasma samples were subsequently collected at surgery in both groups: *n* = 9 no DCA pre-surgery and *n* = 17 with DCA pre-surgery.

**Figure 4.**
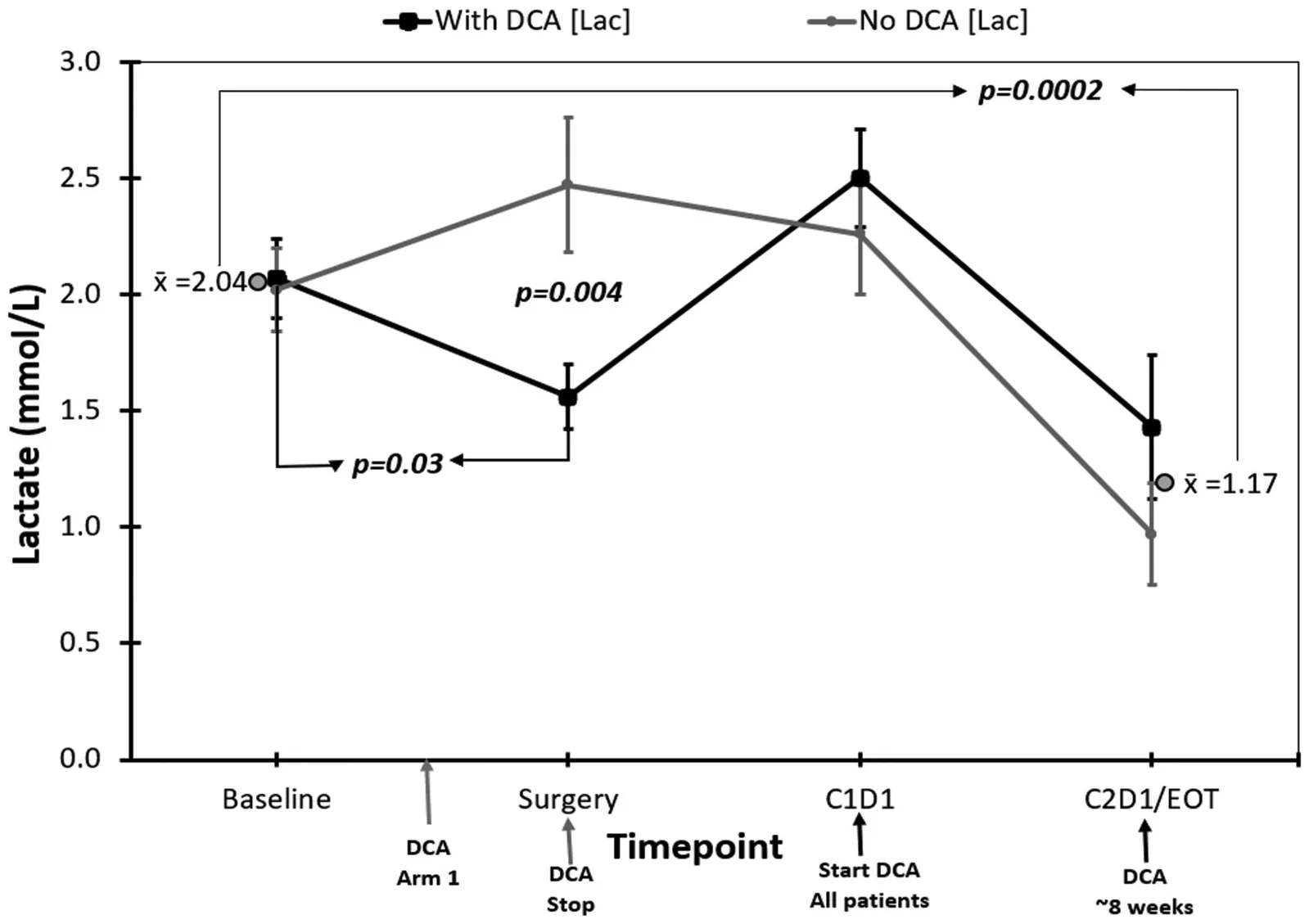
Plasma lactate changes +/− DCA. [Lac]=lactate concentration, C1D1 = Cycle 1 Day 1, C2D1 = Cycle 2 Day 1, EOT = End of treatment.

Lactate levels at baseline were similar between the two arms (2.02 and 2.07 mmol/l, *P* = .84). At surgery, DCA-treated patients had a mean plasma DCA level of 33 ug/mL and lower plasma lactate (1.56 vs 2.47 mmol/l, *P* = .004), compared to those who did not receive preoperative DCA. DCA was not given in the immediate postoperative period; consequently, plasma lactate levels in both patient cohorts were similar off DCA. After DCA was restarted (∼8 weeks of DCA post-surgery), plasma lactate decreased 46% from baseline (2.04 vs 1.17 mmol/l, *P* = .0002) (Figure 4).

### Safety

This trial used precision dosing in administering DCA to patients. Accordingly, only one of 34 chronically treated subjects suffered a mild peripheral neuropathy thought to be due to DCA. This patient’s neuropathy symptoms began 42 days after DCA started. DCA was then held, and 18 days later he had improved and was restarted at 50% of the original dose. His symptoms recurred after 13 days of DCA, and once DCA was discontinued, his neuropathy again resolved. Otherwise, there were no observed drug-related adverse drug effects. A total of 34 patients were included in the safety evaluation. Three patients (8.8%) had treatment-related DLTs, which included intracranial hemorrhage, peripheral neuropathy, and increased alanine aminotransferase. All related and unrelated toxicities are displayed in Table 2. AST, ALT, and fatigue were the only adverse events; with a > 5% incident rate. A full report of adverse events is provided in the supplementary material.

**Table 2.** All adverse events related to DCA (possible, probable, or definite)

CTCAE	Grade 1	Grade 2	Grade 3	Total (%), *N* = 34
Alanine aminotransferase increased	2	1	0	3 (9%)
Alkaline phosphatase increased	1	0	0	1 (3%)
Aspartate aminotransferase increased	2	0	0	2 (6%)
Dizziness	0	1	0	1 (3%)
Fatigue	1	1	1	3 (9%)
Gait disturbance	0	1	0	1 (3%)
Generalized muscle weakness	0	1	0	1 (3%)
Hyperphosphatemia	1	0	0	1 (3%)
Hypoglycemia	0	1	0	1 (3%)
Hypomagnesemia	1	0	0	1 (3%)
Hypophosphatemia	1	0	0	1 (3%)
Intracranial hemorrhage	0	1	0	1 (3%)
Muscle cramp	1	0	0	1 (3%)
Pain of skin	1	0	0	1 (3%)
Peripheral neuropathy	1	1	1	1 (3%)
Platelet count decreased	1	0	0	1 (3%)

### Outcomes

**Figure 5.**
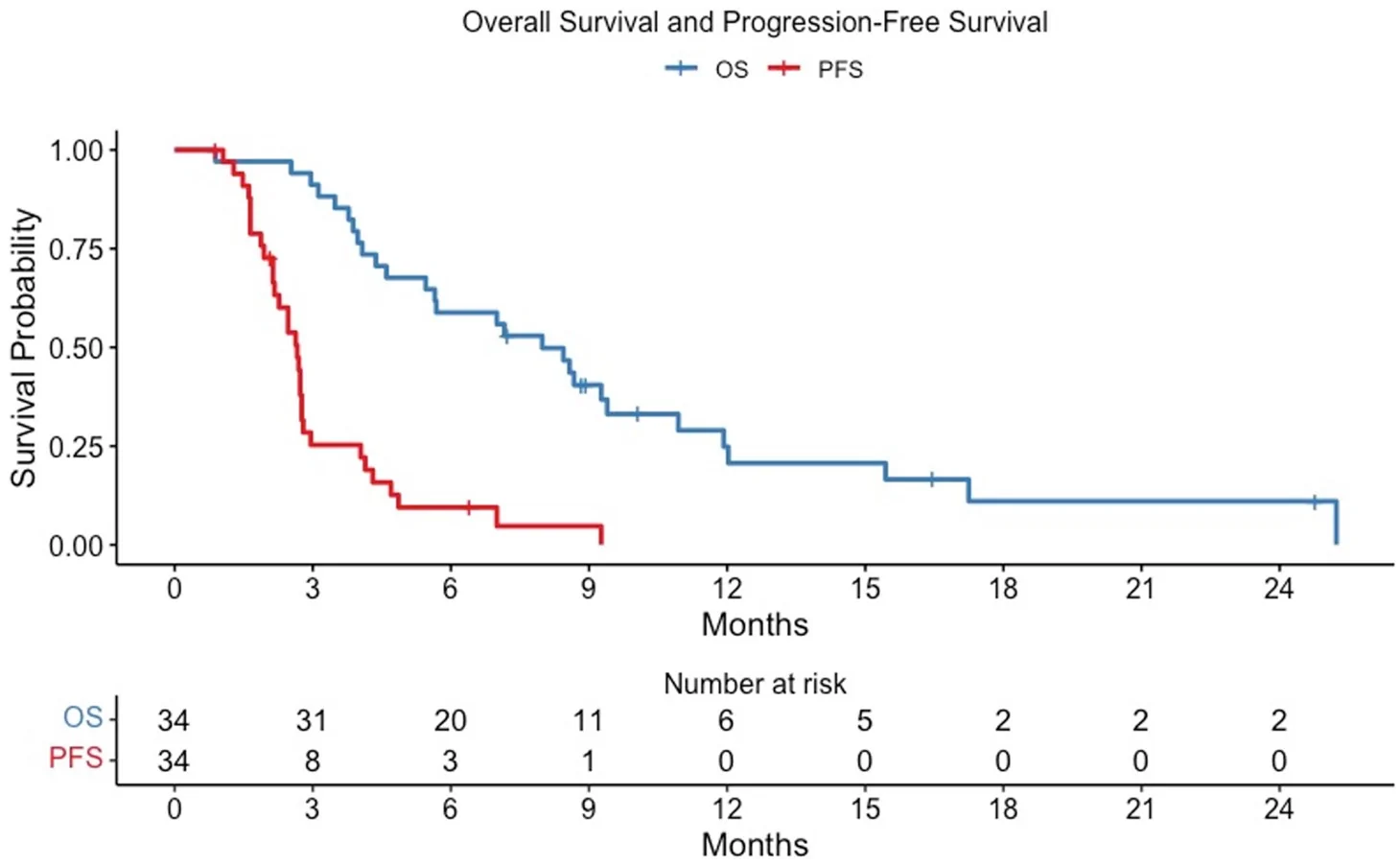
Overall survival and progression-free survival.

A total of 28 (82%) patients had died when the data were analyzed. Twenty-seven patients (79%) had DCA discontinued due to progressive disease. Patients received DCA for only a short period of time due to rapid progression of their disease. The median number of days on treatment was 33 (range 0-112), which was less than one cycle of drug treatment (60 days). The estimated median OS was 8.0 months (95% CI, 4.6-9.4 months) and the estimated median PFS was 2.7 months (95% CI, 2.1-2.8 months) (Figure 5).

## Discussion

For many years, experimental studies have demonstrated that DCA inhibits the activity and/or proliferation of cancer stem cells, including glioma stem cells, principally by shifting metabolism to OXPHOS, resulting in oxidative stress and accelerated apoptosis.^3^^,^^23^ When focused on the PDC/PDK axis as a therapeutic target in brain cancer, DCA has shown evidence of reversing Warburg metabolism, decreasing tumor cell proliferation, and increasing host survival in animal models, and generally good tolerability in small, open-label trials in adults with recurrent GBMs (reviewed in Stacpoole^3^). We surmised that administration of oral DCA 1 week prior to surgery could restore PDC activity and reverse Warburg metabolism in surgically resected tumor tissue from recurrent GBM patients undergoing debulking surgery. Enhancing (T1) and non-enhancing (T2) tumor samples collected at surgery were analyzed separately. The contrast-enhanced tumor tissues are more necrotic and more likely to be anaerobic and depend more on Warburg metabolism, while the non-enhancing tissues are more aerobic and more proliferative.^24^

Although it is reasonable to do so, we did not develop methods to measure tumor tissue levels of DCA. In this study we relied on quantifying changes in such pharmacodynamic endpoints as PDC phosphorylation status and PDK protein expression. In addition, although one or more PDK isoforms are often over-expressed in a pathological state, this was not the case in our GBM patients. Instead, our data indicates that tumor PDC phosphorylation was controlled principally by the PDK4 isoform, reduction of which by DCA led to reactivation of tumor PDC. Furthermore, we have no brain tissue from non-tumor subjects by which to compare PDK protein expressions. Finally, whether peri-surgical conditions (eg anesthesia, duration of surgery, variable tissue oxygenation) influence the expression of certain tumor markers are potential but difficult to control confounding variables of uncertain significance.

In the present study, we demonstrated that oral DCA treatment for 1 week before surgery decreased phosphorylated PDHA1 in the more necrotic enhancing tissue, indicating that DCA can indeed penetrate the tumor tissue and lower p-PDHA1, thereby activating PDC. Phosphorylated PDHA1 was not affected by DCA in the non-enhancing tissue, probably because they are more aerobic and thus metabolically “normal,” compared to the enhancing tissue, which likely depends more on Warburg metabolism.

DCA increases PDC activity by two established mechanisms. The first is by inhibiting the activity of PDKs that inhibit PDC by reversible phosphorylation, thereby maintaining PDHA1 in its unphosphorylated, catalytically active state. The second mechanism by which DCA activates PDC is by stabilizing the complex and decreasing its rate of turnover.^10^^,^^11^ Protein expression of all four PDK isoforms (PDK 1 to 4) were measured, and expression of PDK4 was significantly lower in both enhancing and non-enhancing tumor tissues from patients with DCA pre-surgery, confirming that DCA inhibits PDK, thus, decreasing the levels of the phosphorylated, inactive PDHA1. These findings are consistent with the known action of DCA and suggest a shift toward aerobic respiration. The expression of PDP1, which dephosphorylates and reactivates PDC, was not different with or without DCA in both enhancing and non-enhancing tissue, again consistent with the known action of DCA, ie DCA does not affect PDP1 activity.

The primary difference between brain tumors in general and other solid tumors is the presence of the BBB. The BBB blocks virtually all large molecular weight agents and nearly all small molecules. TMZ has only modest penetration across the BBB (∼ 20%).^25^ Earlier studies have shown that DCA readily crosses the BBB, can be measured in cerebrospinal fluid and enters cells via both the monocarboxylate transporter system and the SLC5A8 plasma membrane transporter that possesses higher affinity for DCA than the monocarboxylate transport system.^9^^,^^10^ Collectively, biochemical data from the present study demonstrate that DCA can cross the BBB and exert its effects on tumor tissues by inhibiting PDK4 in resected tumors from recurrent GBM patients who received DCA 1 week prior to resection surgery and decreased the proportion of the inactive pPDHA1. In turn, this may lead to activation of residual PDC enzyme activity and inhibition of Warburg metabolism. Importantly, the stimulatory effect of DCA on brain PDC activity occurs in both neurons and glia, leading to increased cerebral glucose oxidation *in vivo*. DCA also increases expression of E1α protein in rat liver and in fibroblasts from E1α deficient patients and, to variable extents, that of other components of the PDC complex, regardless of the type of underlying mutation.^26^

A secondary objective of the study was to determine if DCA alters the expression of cell proliferation markers in tumor tissue excised from DCA-treated and DCA-non-treated recurrent GBM patients. HIF1-α and VEGF-α play crucial roles in tumor development and progression, particularly in hypoxia within tumors. HIF1-α acts as a master regulator of cellular response to hypoxia,^27^ while VEGF-α is a key mediator of tumor angiogenesis.^20^ Increased VEGF-α expression is associated with increased tumor growth. In the enhancing, more necrotic tissue, levels of HIF1-α, measured in the nuclear fraction, and VEGF-α were significantly lower in patients who received DCA pre-surgery, indicating that DCA may lower hypoxia-induced angiogenesis in GBM. In the non-contrast tumor tissue, which can be more metabolically active and have more normal vasculature, levels of HIF1-α and VEGF-α were not different with or without DCA. In general, expression of HIF1-α was remarkably higher in the contrast-enhanced tissue compared to the non-contrast-enhanced tissue. Inhibition of the PDC and upregulation of glycolysis by HIFs and other transcription factors (eg Myc) combine to increase intratumoral pyruvate, lactate, and hydrogen ions that stabilize HIF and create a positive feedback loop.^3^ The DCA-associated fall in HIF1-α expression, as observed in the contrast-enhanced tumor from patients who received DCA, may contribute to the decrease in PDK4 and to the decrease in VEGF-α expression,^27^ potentially contributing to the commonly observed inhibitory effects of the drug on angiogenesis, cell proliferation, and tumor growth (reviewed in Stacpoole^3^). Protein expression of phosphoglycerate kinase-1 (PGK1), an essential enzyme in aerobic glycolysis, high expression of which promotes tumor cell proliferation,^19^ was also reduced by DCA in the enhancing tumor. This further indicates that DCA may reduce tumor cell proliferation.

In the non-contrast-enhancing tumor tissue, which can be more proliferative, DCA decreased the levels of the proliferation marker PCNA. PCNA is involved in DNA replication and cell proliferation, and PCNA overexpression is linked to increased tumor cell proliferation and resistance to treatments of DNA repair.^21^ ERK1/2 plays a complex role in cancer development and progression, acting as both a promoter of cancer cell growth and survival and also as a regulator of cell death and senescence.^22^ ERK1/2 activation can induce apoptosis and senescence, and activation of MEK-ERK1/2 signaling is reported to decrease proliferation and migration of glioma cells.^22^^,^^28^ Sustained ERK1/2 activation can also induce apoptosis.^22^ Here, we show that in the more proliferative, non-enhancing tissue, levels of ERK1/2 were increased in tumors from patients who received DCA. The observed increase in ERK1/2 is consistent with prior literature demonstrating context-dependent roles of ERK1/2, including pro-apoptotic effects under certain conditions.

The decrease in PCNA levels is indicative of reduced proliferative activity, while sustained activation of ERK1/2 has been associated with the induction of apoptosis. Taken together, these findings are consistent with a potential antiproliferative effect of DCA in non-enhancing tumor tissue. However, we acknowledge that these markers are not sufficient to definitively establish a direct effect of DCA on tumor cell proliferation. Further studies, including assessment of phospho-ERK1/2 and additional proliferation markers, such as Ki-67, would be required to more conclusively determine whether DCA directly reduces tumor cell proliferation.

The dual mechanism of activation of the PDC by DCA accounts for the drug being the most potent lactate-lowering agent used clinically.^11^ Inhibition of PDKs by DCA reverses Warburg metabolism in cancer cells.^3^ In so doing, it reduces tissue lactate concentrations in both the TME and the blood. Magnetic resonance imaging studies of GBMs have revealed high lactate concentrations relative to normal brain tissue and an inverse association between blood and tumor lactate and survival.^7^ In our study cohort, DCA significantly reduced plasma lactate at surgery in patients who received DCA compared to patients without DCA. When all patients received DCA for up to about 8 weeks post-surgery, plasma lactate levels significantly dropped to almost half, compared to their mean lactate levels at baseline. The decrease in lactate levels correlates with the plasma DCA levels.

A potential mechanism accounting for the inhibitory action of DCA on tumor size is its ability to reduce lactate and H + levels within the TME. Experimental induction of an acidic TME up-regulates expression of proangiogenic and proinflammatory proteins, matrix metalloproteinases, and other peptides associated with enhanced invasive and metastatic potential.^29^ Lactate accumulation in and around tumors *in vivo* has also been associated with metastatic spread and poor clinical outcome.^29^ Lactic acid activates the IL-23/IL-17 pathway and increases arginase-1 in macrophages, which inhibits T-cell proliferation and activation.^30^ The acidic TME leads subsequently to impaired lymphocyte cytotoxicity,^30^ dendritic cell function^31^ and other effects^32^ that, together, dampen the host’s response to the tumor and facilitate invasion and metastasis. Rerouting glucose metabolism with DCA also improves T cell metabolism and their therapeutic efficacy in cancer cells^33^ and has generally beneficial effects in human gliomas.^34^ Nevertheless, efforts to reduce lactate levels in the TME have, to date, focused almost entirely on interrupting lactate production or transport^35^ rather than facilitating its oxidative removal.

The relation between lactic acid and tumor proliferation is further revealed by the finding that experimental reversal of tumor acidity by alkalization inhibits metastatic spread.^36^ DCA stimulation of the PDC leads to increased production of CO_2_ that can be converted to bicarbonate by carbonic anhydrase, consequences that increase the host’s natural buffering capacity and provide a potential mechanism by which the drug inhibits inflammatory mediators. Thus, the effect of DCA on tumor and peri-tumor lactate and H + concentrations probably contributes to the increased survival of DCA-treated animals harboring implanted human cancers, regardless of germ line origin.^27^^,^^37^^,^^38^ Recent findings show that histone lactylation and lactylation-mediated gene expression^35^^,^^39^^,^^40^ disrupt PDC assembly and facilitate tumor progression (including gliomas and glioma stem cells) and chemotherapy resistance.^7^^,^^41-47^ This process is accelerated by hypoxia and emphasizes the interrelationship between metabolism and epigenetics, in which the PDC plays a major role.^48^

Lactate also regulates tumor dynamics by inhibiting cancer cell differentiation and maintaining a stem cell state. Lactate enhances histone acetylation and lactylation, epigenetically activating the Myc oncogene in cells^49^ and enhancing tumor immunosuppression. In contrast, DCA epigenetically promotes differentiation of cancer stemness and, hence, immunotherapy of tumors, by switching tumor cell metabolism from aerobic glycolysis to OXPHOS.^33^ Enhanced mitochondrial respiration drives differentiation of GBM cells into astrocytes.^44^ Accordingly, therapeutic targeting of Warburg metabolism in GBM may yield benefits beyond that achieved with standard chemotherapy.^3^

In conclusion, there was no evidence that DCA affected tumor progression or survival in this small cohort of patients. Indeed, DCA exposure in our small cohort of patients is insufficient to draw conclusions about the drug’s clinical efficacy and its possible effects on survival. However, we did demonstrate that chronic administration of oral DCA to patients with recurrent GBM was well tolerated and safe. Although DCA alone may be insufficient to alter the clinical course of GBM, it may be a useful agent when combined with current standard of care treatment, namely surgery, TMZ, and radiation. In a recently completed federally funded multicenter phase 3 trial, genetics-based dosing of oral DCA administered to 34 young children with congenital PDC deficiency was found to be well-tolerated and safe up to 4 years of continuous treatment, during which it significantly improved morbidity, plasma lactate levels, and survival (manuscript in press), leading to a New Drug Application to the FDA by Saol Therapeutics. DCA is on the threshold of becoming a unique metabolic modulator of PDC in which it is perturbed by genetic or acquired diseases. Accordingly, we intend to continue the studies described here by conducting a phase 2B trial of DCA, combined with standard of care treatment, in newly diagnosed GBM patients to evaluate the potential impact of such combination therapy on patient survival.

## Supplementary Material

vdag223_Supplementary_Data

## Data Availability

All data supporting the findings of this study are available from the corresponding author on reasonable request.
