# Supplementary material for: A novel approach to reverse Warburg metabolism in patients with recurrent glioblastoma: A phase II pharmacodynamic study of dichloroacetate
Source: Neurooncol Adv. 2026 Aug 25;8(1):vdag223. doi: 10.1093/noajnl/vdag223 (PMC13557212; doi:10.1093/noajnl/vdag223)
Supplement: Supplementary file 1 [file vdag223_supplementary_data.docx]

**A novel approach to reverse Warburg metabolism in patients with recurrent glioblastoma: A phase II pharmacodynamic study of dichloroacetate**

**SUPPLEMENTAL MATERIALS**

ELIGIBILITY CRITERIA

1. Patients must be 18-80 years old
2. Patients must have histologically confirmed WHO grade IV glioma that is progressive or recurrent following at least one prior TMZ plus radiation therapy regimen. Patients with previously low-grade glioma who progressed after radiotherapy and chemotherapy and are biopsied and found to have a GBM are eligible. Patients who are truly unmethylated GBMs do not require prior TMZ.
3. Patients must have recurrent GBM as determined by neuroimaging and some degree of symptomatology (e.g., headache, mental status change, seizure), and have clinically indicated tumor debulking surgery planned
4. Patients must have recovered (<CTCAE grade 2 or baseline) from severe toxicity of prior therapy. Patient may have unlimited prior therapies. The following intervals from previous treatments are required to be eligible:
   - 12 weeks from the completion of radiation.
   - 6 weeks from a nitrosourea chemotherapy
   - 3 weeks from a non-nitrosourea chemotherapy
   - 4 weeks from any investigational (not FDA-approved) agents
   - 2 weeks from administration of a non-cytotoxic, FDA-approved agent (e.g., erlotinib, hydroxychloroquine, etc.)
5. Must have completed Standard of Care RT and TMZ. Patients must have received at least 80% of planned TMZ/RT treatment. Patients who are truly unmethylated GBMs do not require prior TMZ patients must have a Karnofsky Performance (KPS) Status

60% (i.e. the patient must be able to care for himself/herself with occasional help from others)

6. Patients may retain whatever medications they are receiving for other conditions (e.g., hypertension, seizures), except patients requiring insulin or sulfonylurea therapy, these patients will not be eligible
7. Women of childbearing potential must have a negative serum pregnancy test within 7 days of dosing and obtain urine pregnancy tests throughout study. Women of childbearing potential and men must agree to use highly effective contraception prior to study entry, for the duration of study participation, and for at least 30 days after the last dose of study drug
8. Patients must have the following organ and marrow function:
   - Absolute neutrophil count ≥ 1,500/ µL
   - Platelets ≥100,000/ µL
   - Hemoglobin ≥ 9 g/dL
   - Total bilirubin ≤ 2mg/dl
   - AST (SGOT)/ALT (SGPT) ≤ 3 × institutional upper limit of normal  GFR > 30ml/min
9. Patients must be able to undergo MRI of the brain with gadolinium. Patients must be maintained on a stable or decreasing dose of corticosteroid regimen (no increase for 5 days) prior to this baseline MRI.
10. Patients who are diabetic must have a screening hemoglobin A1c (Hgb A1c) level of at least 6.0.
12. Patients must be able to provide written informed consent.
13. Patients must have no concurrent malignancy except curatively treated basal or squamous cell carcinoma of the skin or carcinoma in situ of the cervix, breast, or bladder. Patients with prior malignancies must be disease-free for 3 years.
14. Patients must be able to swallow liquid

INELIGIBILITY CRITERIA

1. Patients receiving any other investigational agents for a brain tumor are ineligible.
2. Patients who have not recovered to < CTCAE grade 2 toxicities apart from alopecia related to prior therapy are ineligible.
3. Patients with uncontrolled intercurrent illness including, but not limited to, ongoing or active infection, symptomatic congestive heart failure, clinically significant cardiac disease, unstable angina pectoris, cardiac arrhythmia, or psychiatric illness/social situations that would limit compliance with study requirements, are ineligible.
4. Pregnant women are excluded from this study because the effects of DCA on a fetus are unknown. Because there is an unknown but potential risk for adverse events in nursing infants secondary to treatment of the mother with DCA, breastfeeding should be discontinued if the mother is treated with DCA.
5. Patients with Hgb A1c level more than 6.0 at screening.
